# Supplementary material for: Genetically Defined Organoid Models Reveal Mechanisms Driving Squamous Cell Neoplastic Evolution and Identify Potential Therapeutic Vulnerabilities
Source: bioRxiv. 2025 Jan 22:2025.01.18.631624. Preprint. [Version 1] doi: 10.1101/2025.01.18.631624 (PMC11785044; doi:10.1101/2025.01.18.631624)
Supplement: 1 [file NIHPP2025.01.18.631624V1-supplement-1.pdf]

bioRxiv preprint doi: https://doi.org/10.1101/2025.01.18.631624; this version posted January 22, 2025. The copyright holder for this preprint (which was not certified by peer review) is the author/funder, who has granted bioRxiv a license to display the preprint in perpetuity. It is made available under aCC-BY 4.0 International license.

SUPPLEMENTS

Figure. S1-16

bioRxiv preprint doi: https://doi.org/10.1101/2025.01.18.631624; this version posted January 22, 2025. The copyright holder for this preprint (which was not certified by peer review) is the author/funder, who has granted bioRxiv a license to display the preprint in perpetuity. It is made available under aCC-BY 4.0 International license.

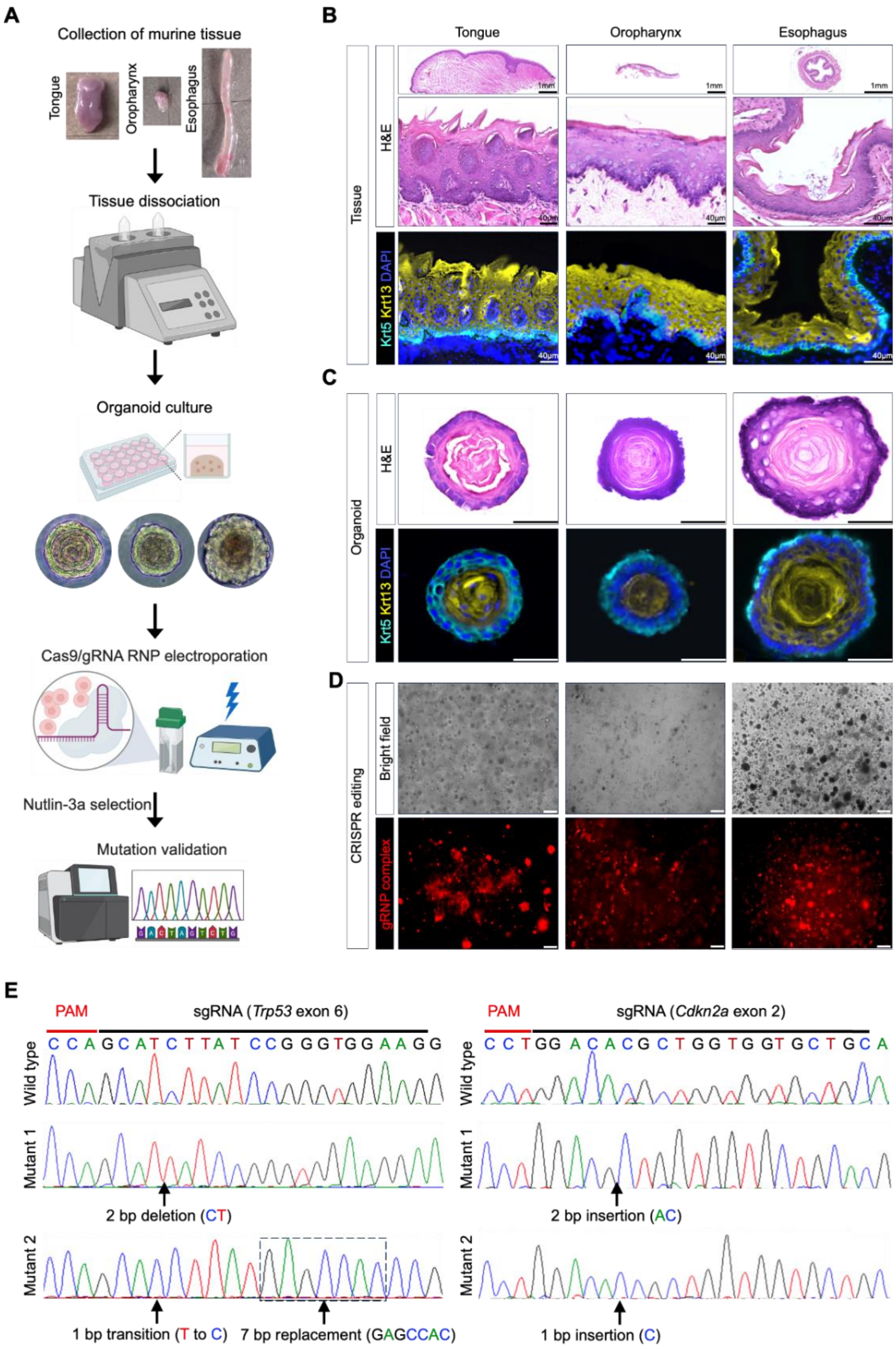

bioRxiv preprint doi: https://doi.org/10.1101/2025.01.18.631624; this version posted January 22, 2025. The copyright holder for this preprint (which was not certified by peer review) is the author/funder, who has granted bioRxiv a license to display the preprint in perpetuity. It is made available under aCC-BY 4.0 International license.

Figure S1. Establishment of CRISPR-Cas9 knockout organoid lines from murine upper aerodigestive tissues. **(A)** Workflow for generating and validating CRISPR-Cas9 edited organoids from mouse tissue. (**B)** Representative H&E and IF images showing staining for the basal cell marker Krt5 (aqua) and the squamous differentiation marker Krt13 (yellow) in mouse tissues. **(C)** Representative H&E and IF images showing staining for Krt5 (aqua) and Krt13 (yellow) in mouse organoids. **(D)** Organoids transfected with Cas9 nuclease and either a negative control gRNA or *Trp53*/*Cdkn2a*-targeted gRNA complex, visualized by red fluorescence. Scale bar, 100 μm. **(E)** Sanger sequencing showing representative mutations at targeted sites, with PAM sequences underlined in red in wild-type sequences. Scale bar, 100 μm.

bioRxiv preprint doi: https://doi.org/10.1101/2025.01.18.631624; this version posted January 22, 2025. The copyright holder for this preprint (which was not certified by peer review) is the author/funder, who has granted bioRxiv a license to display the preprint in perpetuity. It is made available under aCC-BY 4.0 International license.

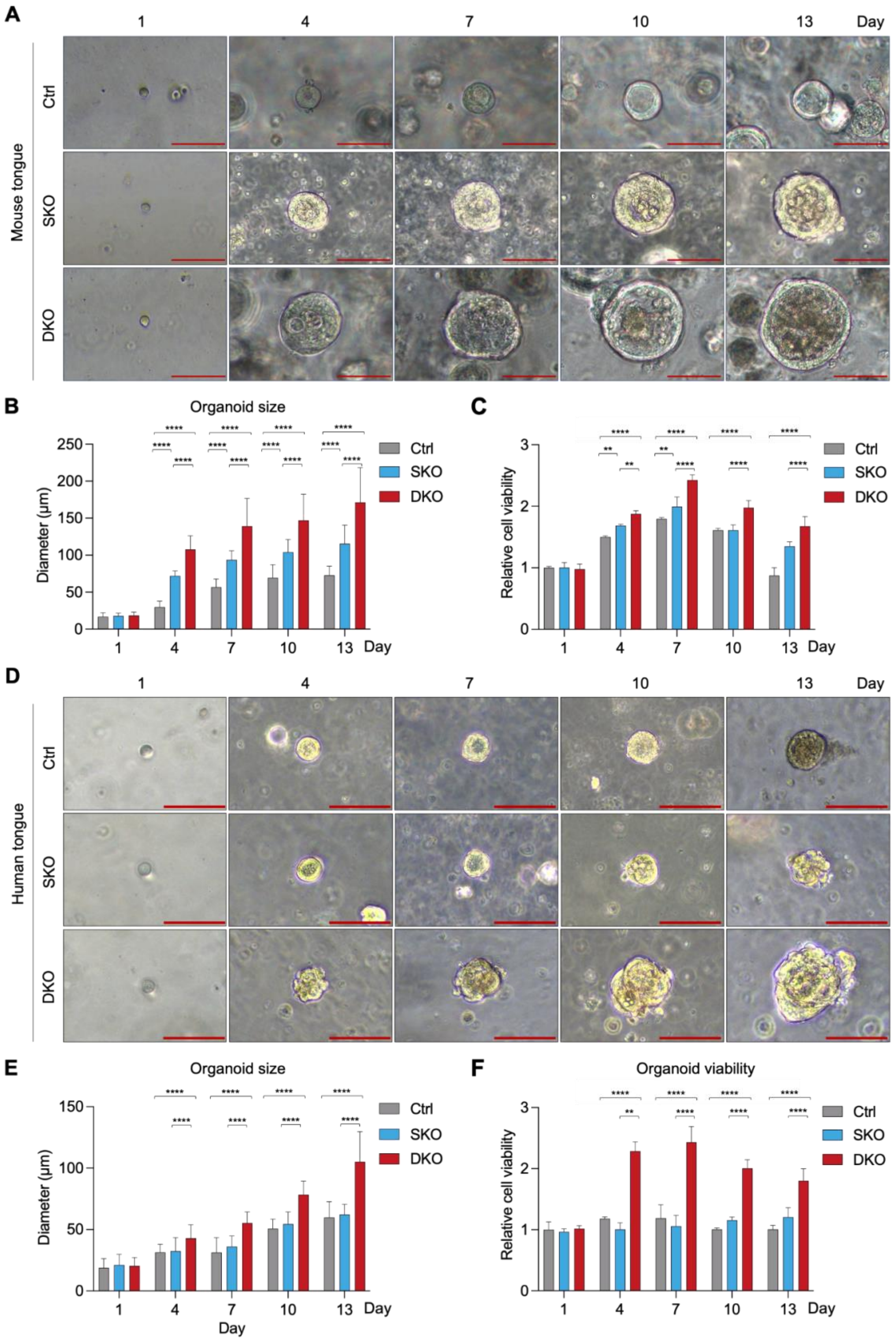

bioRxiv preprint doi: https://doi.org/10.1101/2025.01.18.631624; this version posted January 22, 2025. The copyright holder for this preprint (which was not certified by peer review) is the author/funder, who has granted bioRxiv a license to display the preprint in perpetuity. It is made available under aCC-BY 4.0 International license.

Figure S2. Growth kinetics of genetically-engineered mouse and human tongue organoid lines. (A-C) Mouse tongue Ctrl, SKO and DKO organoids were analyzed for growth properties at the indicated time points: **(A)** Representative phase-contrast photomicrographs of mouse organoid lines. **(B)** Average size of mouse organoids measured at each time point (n = 50 per group). **(C)** Viability of mouse organoids assessed by the WST-1 assay (n = 6 per group). **(D-F)** Human tongue Ctrl, SKO and DKO organoids were analyzed for growth properties at the indicated time points: **(D)** Representative phase-contrast photomicrographs of human organoid lines. **(E)** Average size of human organoids measured at each time point (n = 50 per group). **(F)** Viability of human organoids assessed by the WST-1 assay (n = 6 per group). Scale bar, 100 μm. **$P < 0.01$; ****$P < 0.0001$.

bioRxiv preprint doi: https://doi.org/10.1101/2025.01.18.631624; this version posted January 22, 2025. The copyright holder for this preprint (which was not certified by peer review) is the author/funder, who has granted bioRxiv a license to display the preprint in perpetuity. It is made available under aCC-BY 4.0 International license.

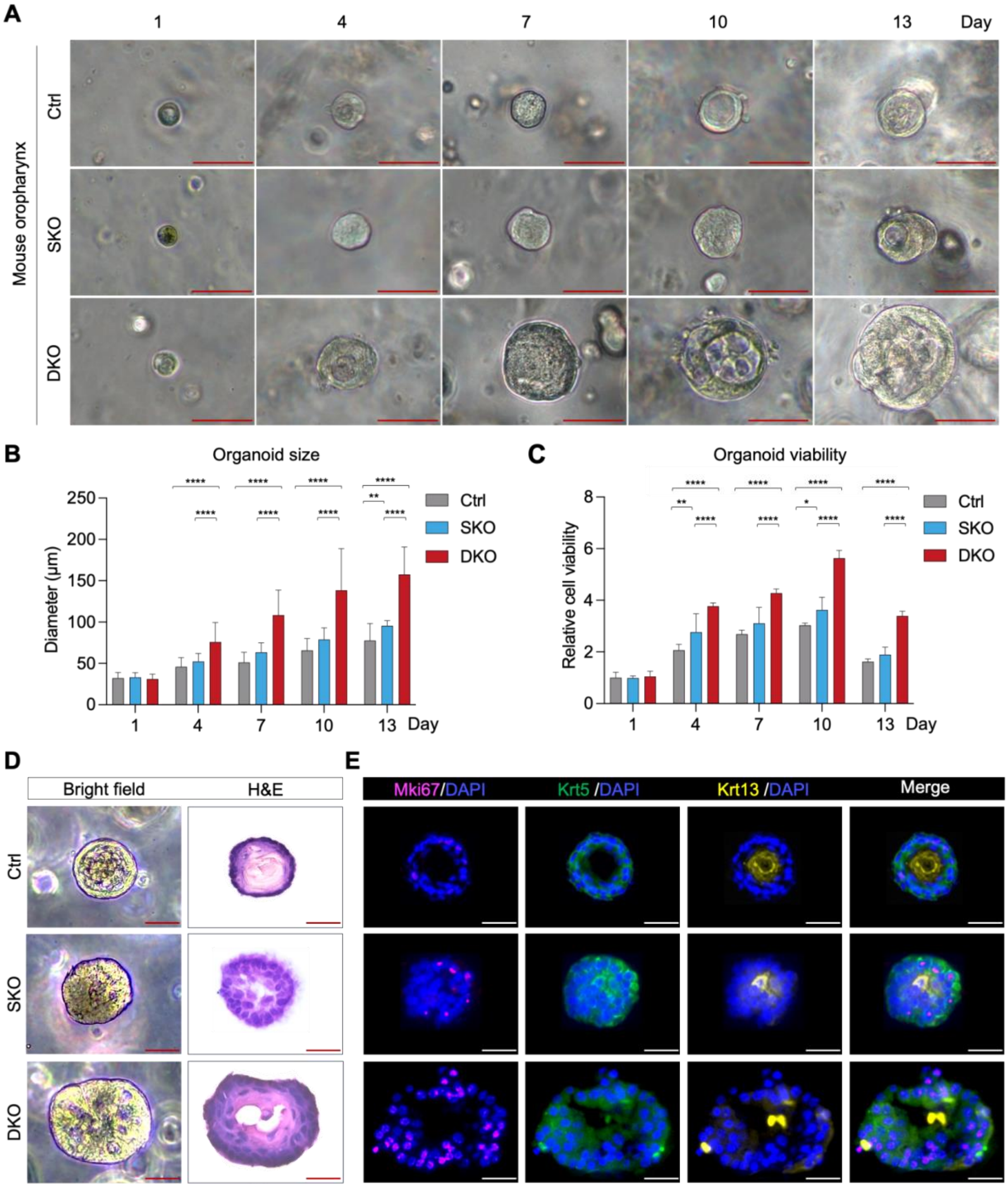

Figure S3. Growth kinetics of genetically-engineered mouse oropharynx organoid lines. (A) Representative phase-contrast photomicrographs of mouse oropharynx organoid lines. **(B)** Average size of mouse oropharynx organoid lines measured at each time point (n = 50 per group). **(C)** Viability of mouse oropharynx organoid lines assessed by the WST-1 assay (n = 6 per group).

bioRxiv preprint doi: https://doi.org/10.1101/2025.01.18.631624; this version posted January 22, 2025. The copyright holder for this preprint (which was not certified by peer review) is the author/funder, who has granted bioRxiv a license to display the preprint in perpetuity. It is made available under aCC-BY 4.0 International license.

(**D)** Representative bright-field, H&E, and (**E**) IF images showing staining for the proliferation marker Mki67 (magenta), basal cell marker Krt5 (green), and squamous differentiation marker Krt13 (yellow) in 3-week-old mouse oropharynx Ctrl, SKO, and DKO organoids. Scale bar, 100 μm. *P < 0.05; **P < 0.01; ****P < 0.0001.

bioRxiv preprint doi: https://doi.org/10.1101/2025.01.18.631624; this version posted January 22, 2025. The copyright holder for this preprint (which was not certified by peer review) is the author/funder, who has granted bioRxiv a license to display the preprint in perpetuity. It is made available under aCC-BY 4.0 International license.

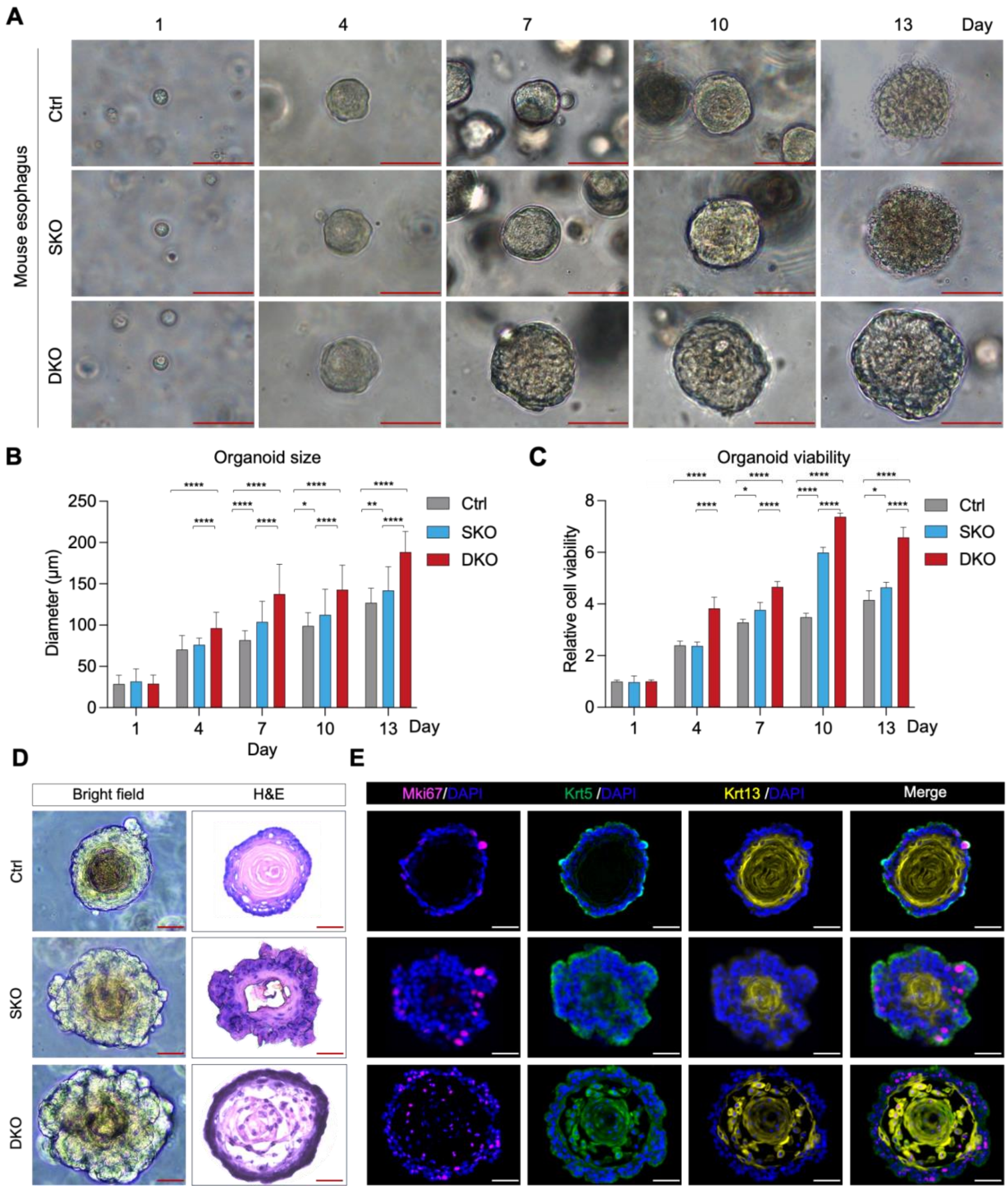

Figure S4. Growth kinetics of genetically-engineered mouse esophageal organoid lines. (A) Representative phase-contrast photomicrographs of mouse esophageal organoid lines. **(B)** Average size of mouse esophageal organoid lines measured at the indicated time points (n = 50 per group). **(C)** Viability of mouse oropharynx organoid lines assessed by the WST-1 assay (n =

bioRxiv preprint doi: https://doi.org/10.1101/2025.01.18.631624; this version posted January 22, 2025. The copyright holder for this preprint (which was not certified by peer review) is the author/funder, who has granted bioRxiv a license to display the preprint in perpetuity. It is made available under aCC-BY 4.0 International license.

6 per group). **(D)** Representative bright-field, H&E, and **(E)** IF images showing staining for the proliferation marker Mki67 (magenta), basal cell marker Krt5 (green), and squamous differentiation marker Krt13 (yellow) in 3-week-old mouse esophageal Ctrl, SKO, and DKO organoids. Scale bar, 100 μm. $^*P < 0.05$; $^{**}P < 0.01$; $^{****}P < 0.0001$.

bioRxiv preprint doi: https://doi.org/10.1101/2025.01.18.631624; this version posted January 22, 2025. The copyright holder for this preprint (which was not certified by peer review) is the author/funder, who has granted bioRxiv a license to display the preprint in perpetuity. It is made available under aCC-BY 4.0 International license.

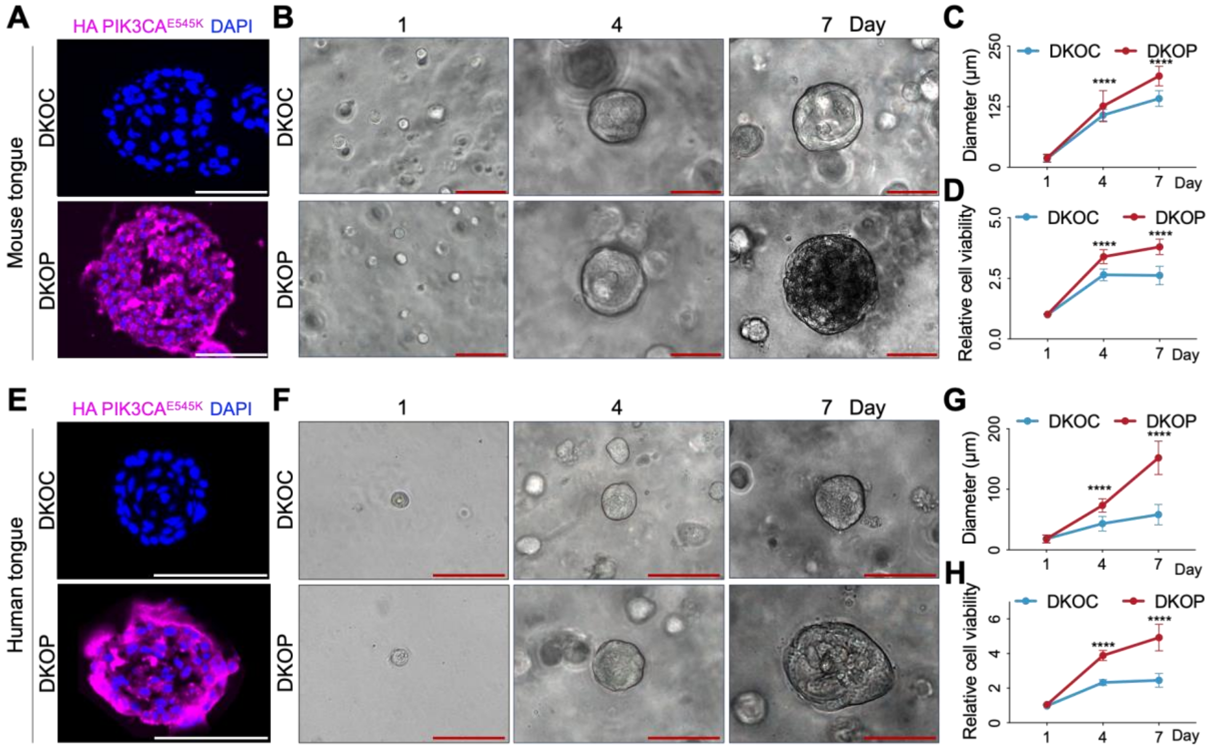

Figure S5. Analysis of PIK3CA^{E545K} expression and growth properties in mouse and human tongue DKOC and DKOP organoids. (A-D) Mouse tongue DKOC and DKOP organoids: **(A)** IF staining images showing HA-PIK3CA^{E545K} (magenta) expression in mouse organoid lines. **(B)** Representative phase-contrast photomicrographs of mouse organoid lines. **(C)** Average size of mouse organoid lines measured at the indicated time points (n = 50 per group). **(D)** Viability of mouse organoid lines assessed by the WST-1 assay (n = 6 per group). **(E-H)** Human tongue DKOC and DKOP organoids: **(E)** IF staining images showing HA-PIK3CA^{E545K} (magenta) expression in human organoid lines. **(F)** Representative phase-contrast photomicrographs of human organoid lines. **(G)** Average size of human organoid lines measured at the indicated time points (n = 50 per group). **(H)** Viability of human organoid lines assessed by the WST-1 assay (n = 6 per group). Scale bar, 100 μm. ****$P < 0.0001$.

bioRxiv preprint doi: https://doi.org/10.1101/2025.01.18.631624; this version posted January 22, 2025. The copyright holder for this preprint (which was not certified by peer review) is the author/funder, who has granted bioRxiv a license to display the preprint in perpetuity. It is made available under aCC-BY 4.0 International license.

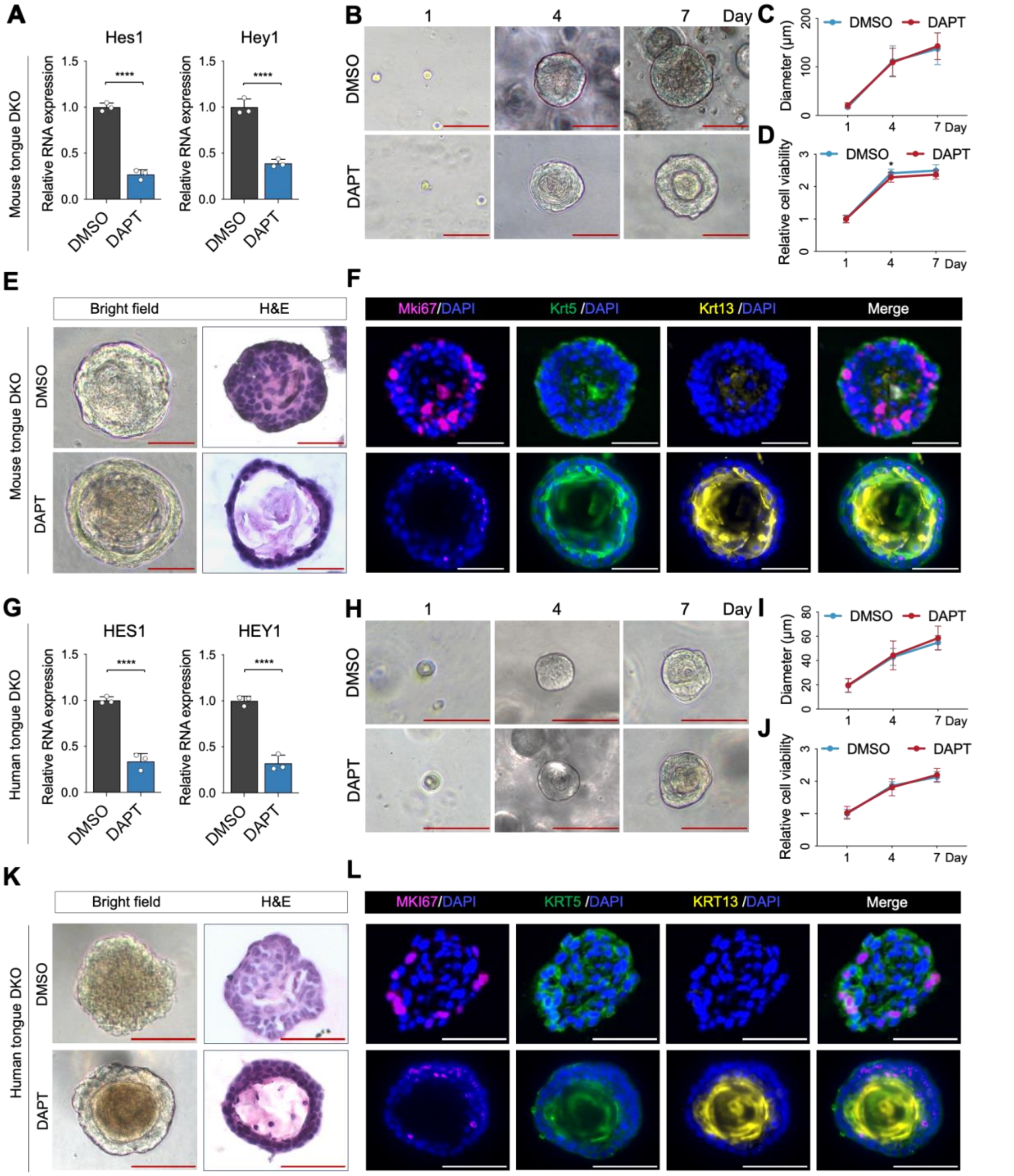

Figure S6. **Analysis of NOTCH inhibition and growth properties in human tongue DKOC and DKOP organoids. (A-F)** Mouse tongue DKO organoids treated with DMSO or the NOTCH inhibitor DAPT: **(A)** Relative mRNA expression levels of key Notch signaling-related genes. **(B)** Representative phase-contrast photomicrographs of mouse organoid lines. **(C)** Average size of

bioRxiv preprint doi: https://doi.org/10.1101/2025.01.18.631624; this version posted January 22, 2025. The copyright holder for this preprint (which was not certified by peer review) is the author/funder, who has granted bioRxiv a license to display the preprint in perpetuity. It is made available under aCC-BY 4.0 International license.

mouse organoids measured at the indicated time points (n = 50 per group). **(D)** Viability of mouse organoids assessed using the WST-1 assay (n = 15 per group). **(E)** Representative bright-field and H&E images, and **(F)** IF images showing staining for the proliferation marker Mki67 (magenta), basal cell marker Krt5 (green), and squamous differentiation marker Krt13 (yellow) in 3-week-old mouse organoids. **(G-L)** Human tongue DKO organoids treated with DMSO or DAPT: **(G)** Relative mRNA expression levels of key NOTCH signaling-related genes. **(H)** Representative phase-contrast photomicrographs of human organoid lines. **(I)** Average size of human organoids measured at the indicated time points (n = 50 per group). **(J)** Viability of human organoids assessed using the WST-1 assay (n = 15 per group). **(K)** Representative bright-field and H&E images, and **(L)** IF images showing staining for the proliferation marker MKI67 (magenta), basal cell marker KRT5 (green), and squamous differentiation marker KRT13 (yellow) in 3-week-old human organoids. Scale bar, 100 μm. ****P < 0.0001.

bioRxiv preprint doi: https://doi.org/10.1101/2025.01.18.631624; this version posted January 22, 2025. The copyright holder for this preprint (which was not certified by peer review) is the author/funder, who has granted bioRxiv a license to display the preprint in perpetuity. It is made available under aCC-BY 4.0 International license.

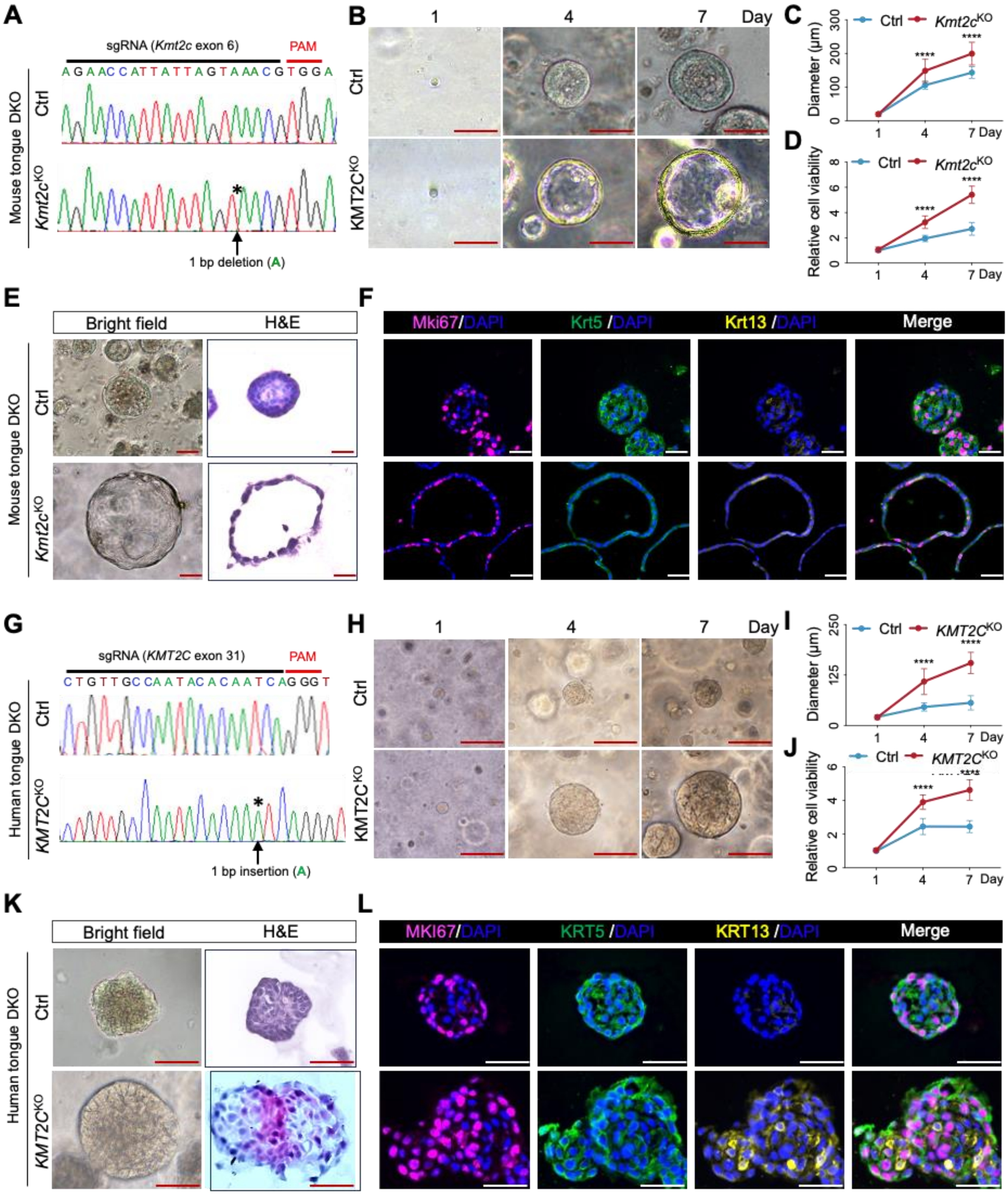

Figure S7. **Analysis of targeted *KMT2C* knockout and growth properties in mouse and human organoids. (A-F)** Mouse tongue DKO organoids with Ctrl (non-targeting sgRNA) or *Kmt2c* knockout: **(A)** Sanger sequencing showing representative mutations at the targeted Kmt2c sites. **(B)** Representative phase-contrast photomicrographs of mouse organoid lines. **(C)** Average size of mouse organoids measured at the indicated time points (n = 50 per group). **(D)** Viability of mouse organoids

bioRxiv preprint doi: https://doi.org/10.1101/2025.01.18.631624; this version posted January 22, 2025. The copyright holder for this preprint (which was not certified by peer review) is the author/funder, who has granted bioRxiv a license to display the preprint in perpetuity. It is made available under aCC-BY 4.0 International license.

assessed using the WST-1 assay (n = 6 per group). **(E)** Representative bright-field and H&E, and **(F)** IF images showing staining for the proliferation marker Mki67 (magenta), basal cell marker Krt5 (green), and squamous differentiation marker Krt13 (yellow) in 3-week-old mouse organoids. **(G-L)** Human tongue DKO organoids with Ctrl or *KMT2C* knockout: **(G)** Sanger sequencing showing representative mutations at the targeted *KMT2C* sites. **(H)** Representative phase-contrast photomicrographs of human organoid lines. **(I)** Average size of human organoids measured at the indicated time points (n = 50 per group). **(J)** Viability of human organoids assessed using the WST-1 assay (n = 6 per group). **(K)** Representative bright-field and H&E images of 3-week-old human organoids. **(L)** IF images showing staining for MKI67 (magenta), KRT5 (green), and KRT13 (yellow) in 3-week-old human organoids. Scale bar, 100 µm. ****$P < 0.0001$.

bioRxiv preprint doi: https://doi.org/10.1101/2025.01.18.631624; this version posted January 22, 2025. The copyright holder for this preprint (which was not certified by peer review) is the author/funder, who has granted bioRxiv a license to display the preprint in perpetuity. It is made available under aCC-BY 4.0 International license.

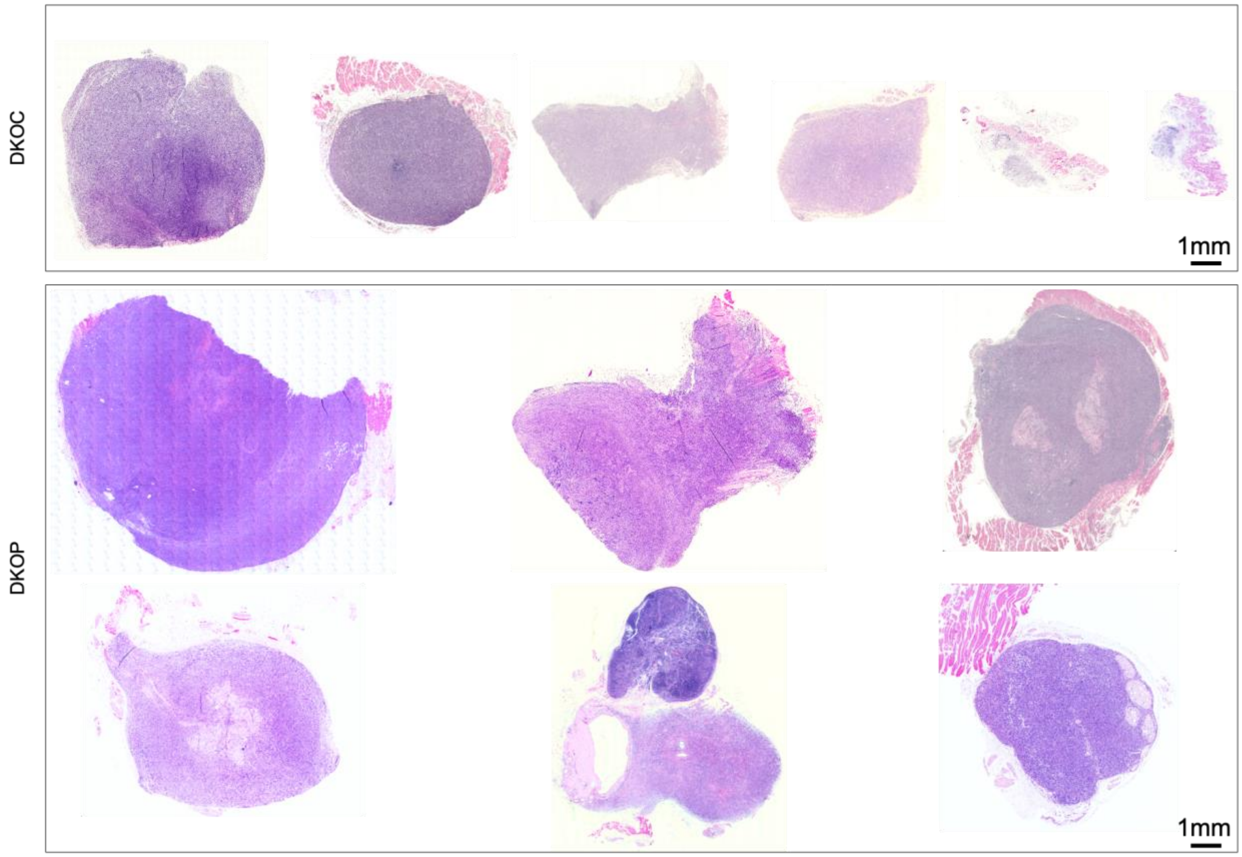

Figure S8. H&E staining of tumors formed by mouse tongue DKOC and DKOP organoids in vivo.

bioRxiv preprint doi: https://doi.org/10.1101/2025.01.18.631624; this version posted January 22, 2025. The copyright holder for this preprint (which was not certified by peer review) is the author/funder, who has granted bioRxiv a license to display the preprint in perpetuity. It is made available under aCC-BY 4.0 International license.

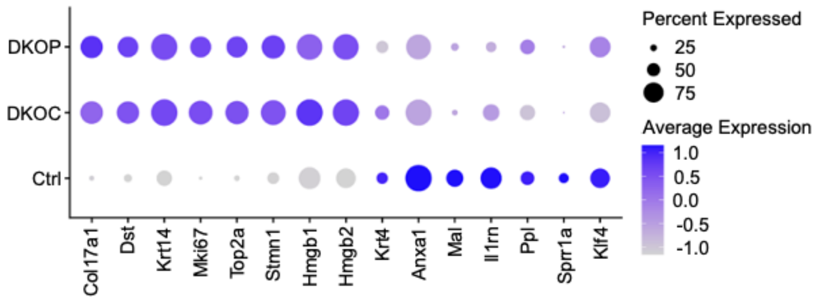

Figure S9. Dot plot showing the expression of selected marker genes in mouse tongue organoid lines.

bioRxiv preprint doi: https://doi.org/10.1101/2025.01.18.631624; this version posted January 22, 2025. The copyright holder for this preprint (which was not certified by peer review) is the author/funder, who has granted bioRxiv a license to display the preprint in perpetuity. It is made available under aCC-BY 4.0 International license.

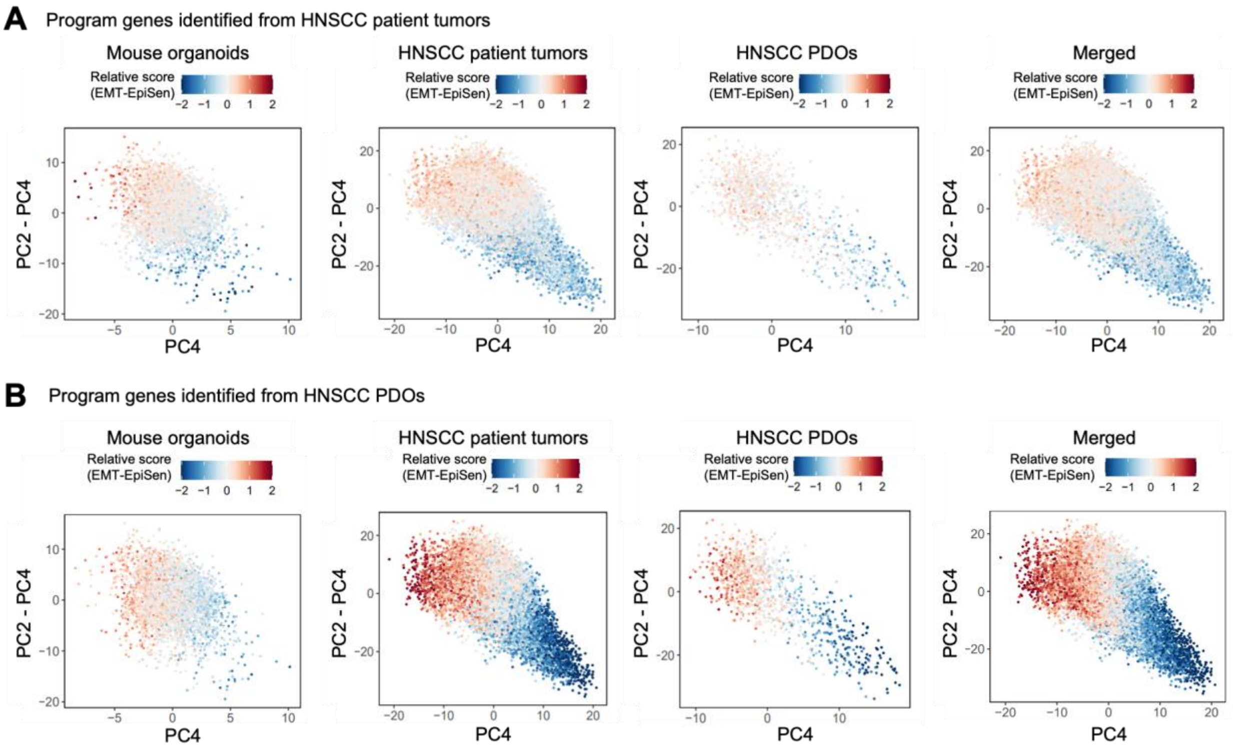

Figure S10. PCA plots of indicated single-cell samples. Cells from mouse organoids, HNSCC patient tumors, and HNSCC PDOs are colored by relative scores for EMT and EpiSen genesets identified in **(A)** HNSCC patient tumors and **(B)** HNSCC PDOs.

bioRxiv preprint doi: https://doi.org/10.1101/2025.01.18.631624; this version posted January 22, 2025. The copyright holder for this preprint (which was not certified by peer review) is the author/funder, who has granted bioRxiv a license to display the preprint in perpetuity. It is made available under aCC-BY 4.0 International license.

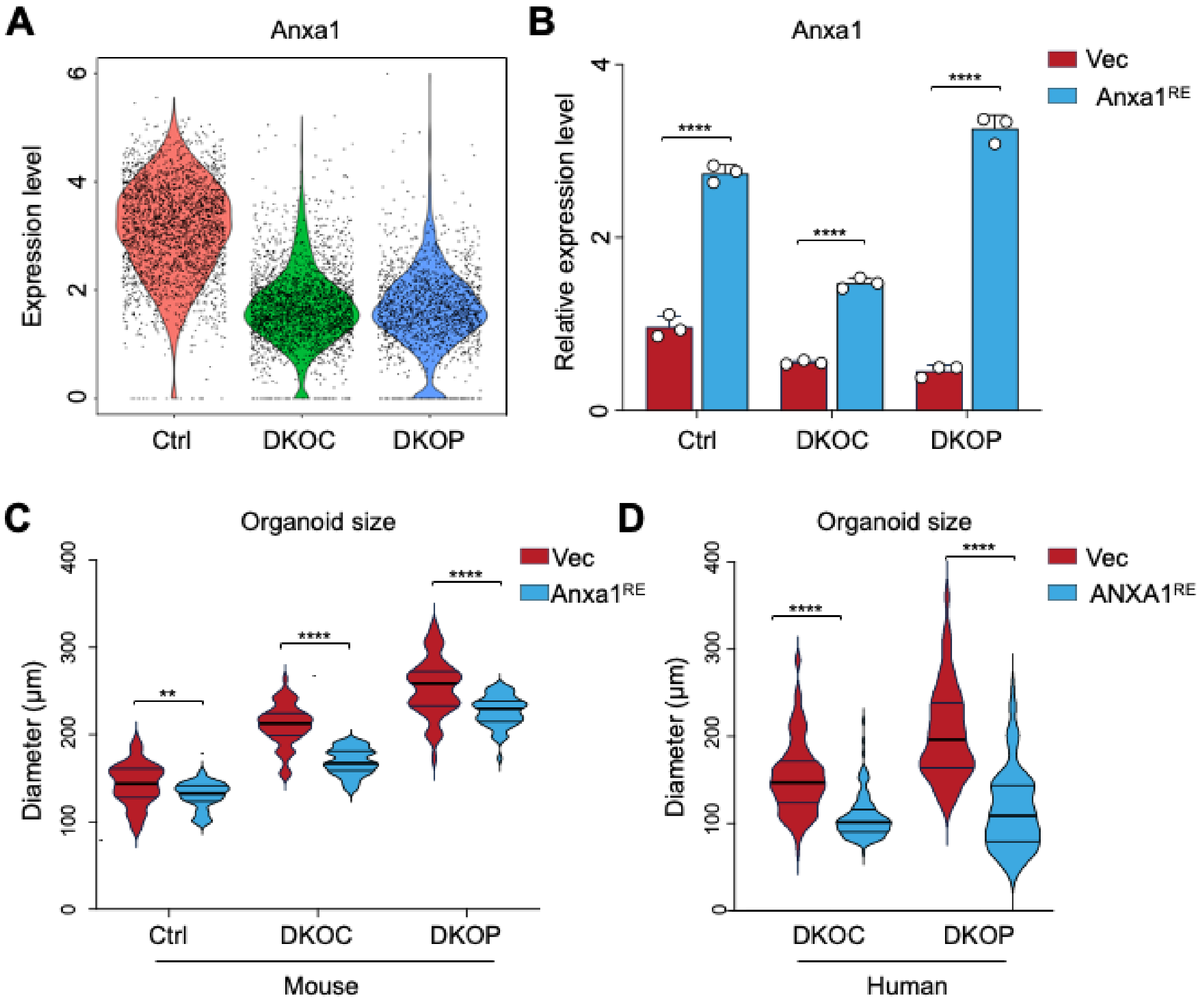

Figure S11. Analysis of ANXA1 expression and growth properties in mouse and human tongue organoids. (A) Anxa1 expression in mouse tongue organoids analyzed using scRNA-seq data. **(B)** Relative Anxa1 mRNA expression in Vec and Anxa1^{RE} mouse tongue organoid lines, normalized to the mouse tongue Ctrl Vec group. **(C)** Average size of mouse and human tongue Ctrl, DKOC, and DKOP organoid lines. ***P* < 0.01; ***P* < 0.0001.

bioRxiv preprint doi: https://doi.org/10.1101/2025.01.18.631624; this version posted January 22, 2025. The copyright holder for this preprint (which was not certified by peer review) is the author/funder, who has granted bioRxiv a license to display the preprint in perpetuity. It is made available under aCC-BY 4.0 International license.

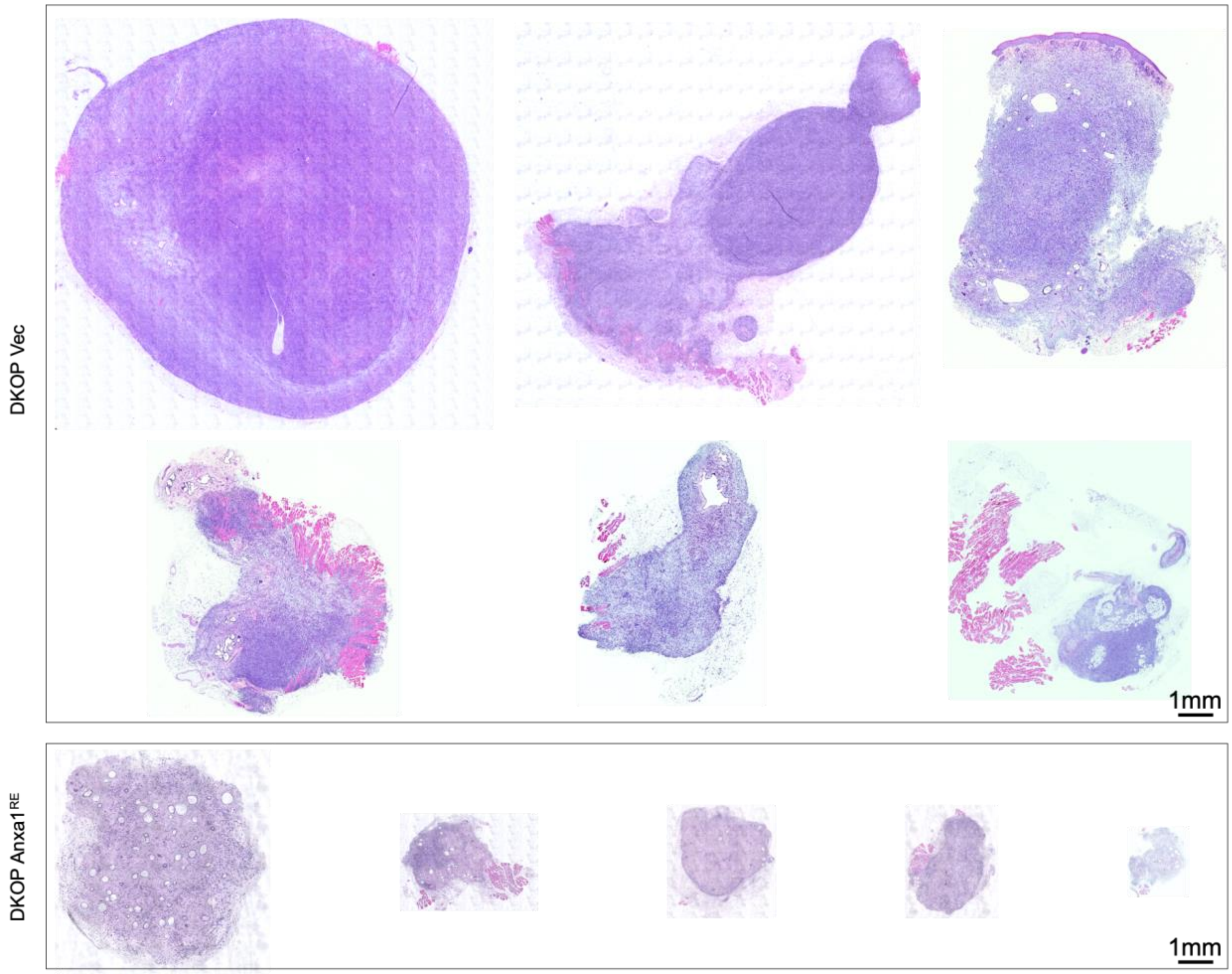

Figure S12. H&E staining showing Anxa1^{RE} in DKOP organoids yields fewer and smaller allografts with reduced atypical features.

bioRxiv preprint doi: https://doi.org/10.1101/2025.01.18.631624; this version posted January 22, 2025. The copyright holder for this preprint (which was not certified by peer review) is the author/funder, who has granted bioRxiv a license to display the preprint in perpetuity. It is made available under aCC-BY 4.0 International license.

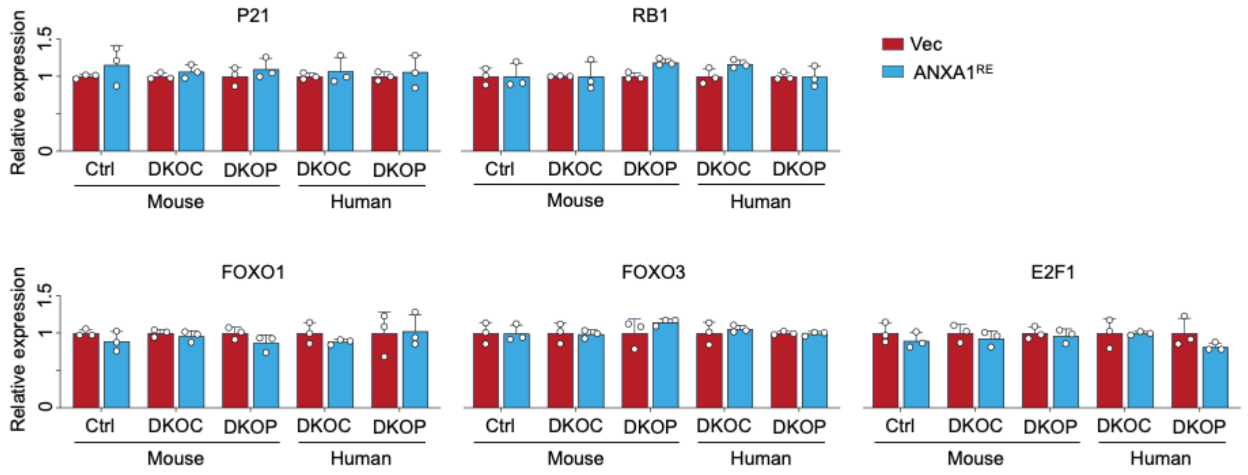

Figure S13. Relative mRNA expression in Vec and Anxa1^{RE} mouse and human tongue organoid lines, normalized to the corresponding Vec group.

bioRxiv preprint doi: https://doi.org/10.1101/2025.01.18.631624; this version posted January 22, 2025. The copyright holder for this preprint (which was not certified by peer review) is the author/funder, who has granted bioRxiv a license to display the preprint in perpetuity. It is made available under aCC-BY 4.0 International license.

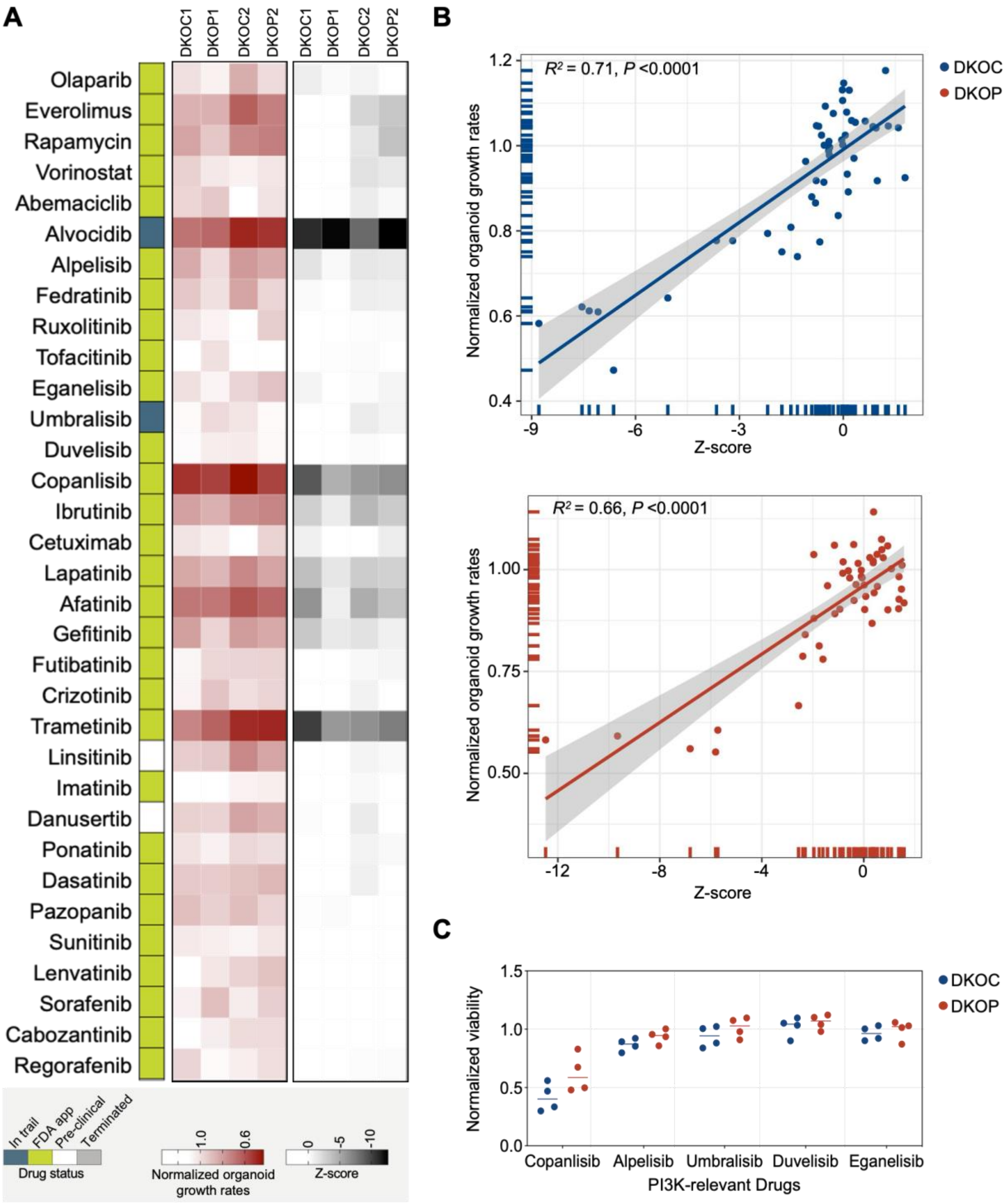

Figure S14. Drug screening and correlation analysis of ATP-based measurements with organoid viability assessed via machine-learning-assisted image analysis. (A) Heatmaps of growth rates and ATP assay results (z-scores) for organoids. Growth rates were calculated as the ratio of organoid growth on day 6 (prior to the ATP assay) to day 4 (before the initial drug

bioRxiv preprint doi: https://doi.org/10.1101/2025.01.18.631624; this version posted January 22, 2025. The copyright holder for this preprint (which was not certified by peer review) is the author/funder, who has granted bioRxiv a license to display the preprint in perpetuity. It is made available under aCC-BY 4.0 International license.

treatment), normalized to the DMSO vehicle control. Data from each of the two independent biological replicates are shown. **(B)** Scatter plot of linear regression analysis between z-score and organoid growth rates assessed through machine learning-based image analysis. **(C)** Viability of mouse DKOC and DKOP organoids under PI3K-relevant drug treatments, measured by ATP assay.

bioRxiv preprint doi: https://doi.org/10.1101/2025.01.18.631624; this version posted January 22, 2025. The copyright holder for this preprint (which was not certified by peer review) is the author/funder, who has granted bioRxiv a license to display the preprint in perpetuity. It is made available under aCC-BY 4.0 International license.

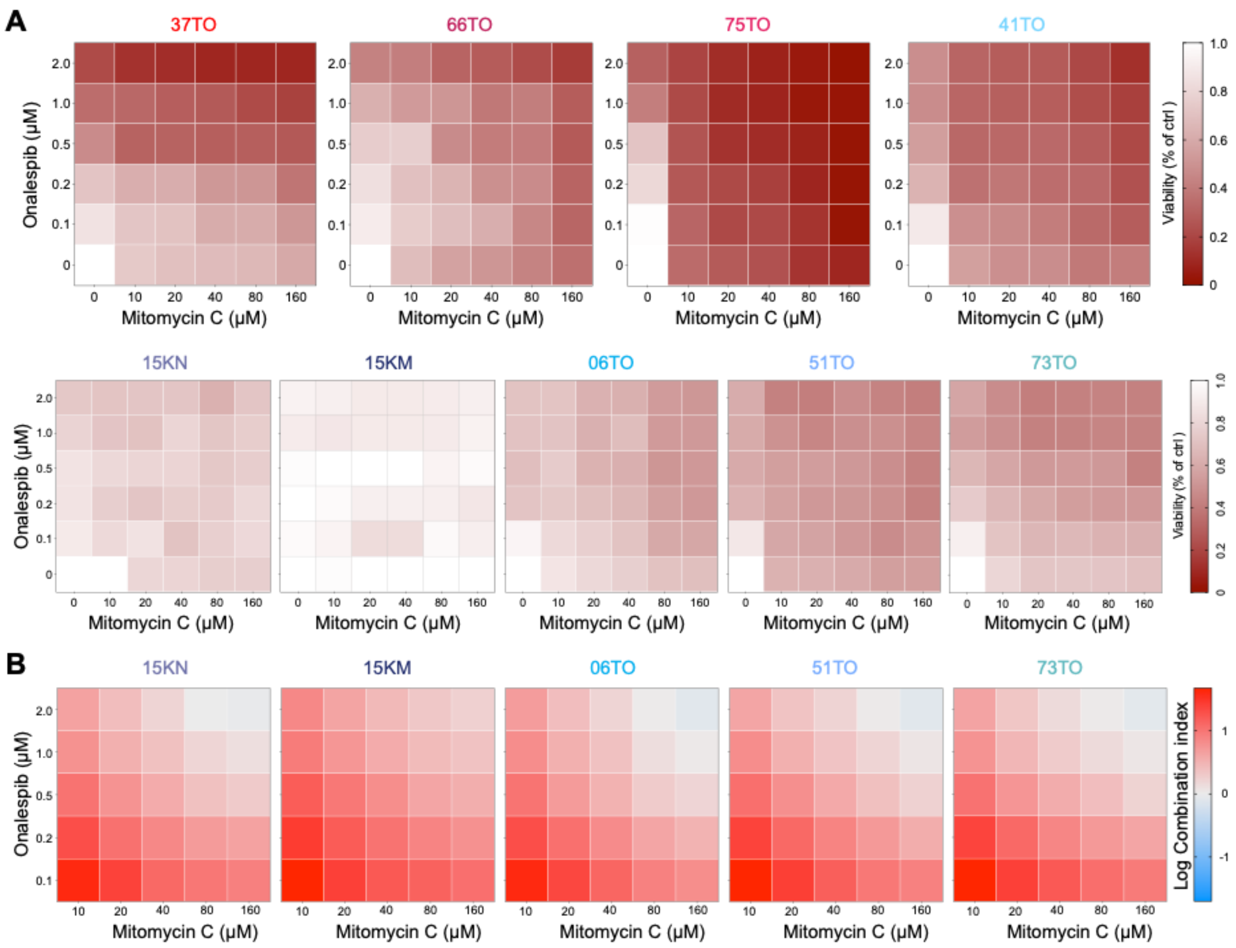

Figure S15. Sensitivity and combination index analysis of Mitomycin C and Onalespib in *PIK3CA* wild-type and mutant PDOs. (A) Sensitivity of *PIK3CA*-mutant (37TO, 66TO, and 75TO), *PIK3CA*-amplified (41TO) and wild-type (lower panel) PDOs to the combination of Mitomycin C and Onalespib (n=3). The data is normalized to DMSO control. **(B)** Combination index (CI) of Mitomycin C and Onalespib across wild-type *PIK3CA* PDOs (n=3). Log CI > 0 indicates antagonism; Log CI = 0 indicates additivity; Log CI < 0 indicates synergy.

bioRxiv preprint doi: https://doi.org/10.1101/2025.01.18.631624; this version posted January 22, 2025. The copyright holder for this preprint (which was not certified by peer review) is the author/funder, who has granted bioRxiv a license to display the preprint in perpetuity. It is made available under aCC-BY 4.0 International license.

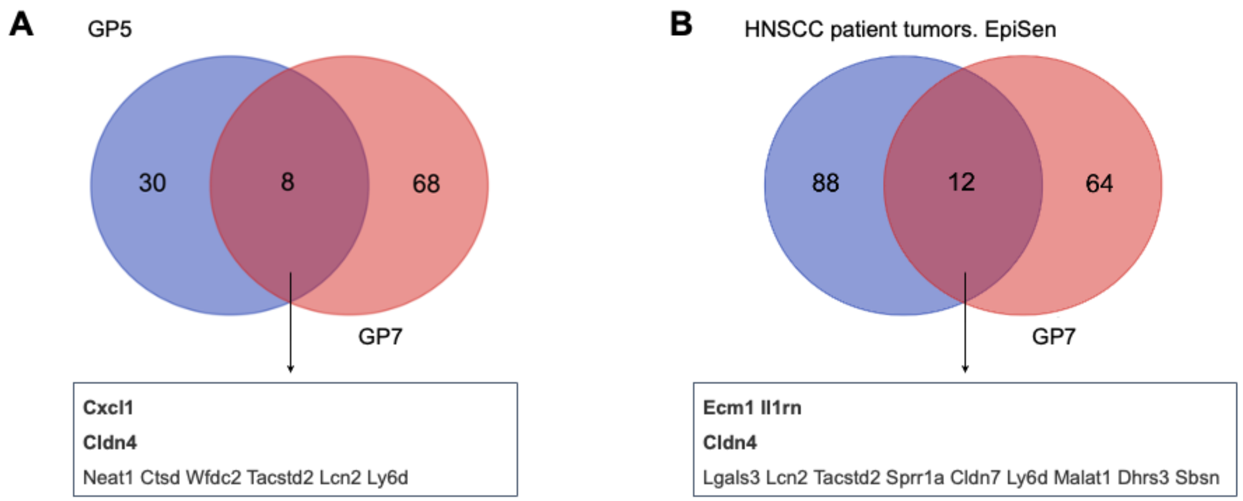

Figure S16. **Venn diagram analysis of overlapping genes between mouse organoid GP7 and GP5 (left), and between GP7 and the EpiSen program in HNSCC patient tumors (right).**

Table. S1-3

Table S1. Clinical information of patient samples

Table S2. Key resources table

Table S3. Gene list for identified GPs
